# Effects of Different Divalent Cation Hydrothermal Treatments of Titanium Implant Surfaces for Epithelial Tissue Sealing

**DOI:** 10.3390/ma13092038

**Published:** 2020-04-27

**Authors:** Xudiyang Zhou, Ikiru Atsuta, Yasunori Ayukawa, Ikue Narimatsu, Tianren Zhou, Jiangqi Hu, Kiyoshi Koyano

**Affiliations:** 1 Section of Implant and Rehabilitative Dentistry, Division of Oral Rehabilitation, Faculty of Dental Science, Kyushu University, Fukuoka 812-8582, Japan; zxdy@dent.kyushu-u.ac.jp (X.Z.); narimatu.i@dent.kyushu-u.ac.jp (I.N.); shu0506@dent.kyushu-u.ac.jp (T.Z.); kokouki@dent.kyushu-u.ac.jp (J.H.); koyano@dent.kyushu-u.ac.jp (K.K.); 2 Division of Advanced Dental Devices and Therapeutics, Faculty of Dental Science, Kyushu University, Fukuoka 812-8582, Japan; atyuta@dent.kyushu-u.ac.jp

**Keywords:** divalent cations, hydrothermal treatment, titanium dental implant, epithelial cells and tissue, Laminin-332

## Abstract

The improvement of peri-implant epithelium (PIE) adhesion to titanium (Ti) may promote Ti dental implant stability. This study aims to investigate whether there is a positive effect of Ti hydrothermally treated (HT) with calcium chloride (CaCl_2_), zinc chloride (ZnCl_2_), and strontium chloride (SrCl_2_) on promoting PIE sealing. We analyzed the response of a rat oral epithelial cell (OEC) culture and performed an in vivo study in which the maxillary right first molars of rats were extracted and replaced with calcium (Ca)-HT, zinc (Zn)-HT, strontium (Sr)-HT, or non-treated control (Cont) implants. The OEC adhesion on Ca-HT and Zn-HT Ti plates had a higher expression of adhesion proteins than cells on the Cont and Sr-HT Ti plates. Additionally, the implant PIE of the Ca-HT and Zn-HT groups revealed better expression of immunoreactive laminin-332 (Ln-322) at 2 weeks after implantation. The Ca-HT and Zn-HT groups also showed better attachment at the implant–PIE interface, which inhibited horseradish peroxidase penetration. These results demonstrated that the divalent cations of Ca (Ca^2+^) and Zn (Zn^2+^)-HT improve the integration of epithelium around the implant, which may facilitate the creation of a soft barrier around the implant to protect it from foreign body penetration.

## 1. Introduction

Titanium (Ti) dental implants are becoming more widely used in edentulous treatments. However, successful oral implant treatments depend on bone contact and the seal between the implant and surrounding gingival tissue [1]. A solid seal between the implant surface and epithelium is necessary to prevent bacterial invasion [2].

A previous study reported that the cell adhesion for epithelial sealing could be affected by different topographies of the implant surface [3]. Hemidesmosome (HD), a multi-protein complex, is a crucial cell adhesion structure that could enable the stable adhesion of epithelial cells to the underlying basement membrane [4,5]. HDs containing laminin-332 (Ln-332) in the basement membrane play a key defensive role against peri-implant tissue breakdown [6,7]. Moreover, Ln-332 could associate intracellularly with plectin, which interacts, in succession, with the keratin filament system [8].

Ti is used as a dental implant material as it has stable mechanical properties and excellent biocompatibility [9]. Numerous Ti implant surface modification techniques accelerate bone contact on implant surfaces by changing the surface topography. Alternatively, previous studies found that a roughened surface had a negative effect on epithelium cell adhesion [10] and that roughened surfaces often promote dental plaque accumulation on an implant surface. Among the many surface modifications of Ti, hydrothermal treatments have been demonstrated to improve bioactivity and osteoconductivity [11]. Hydrothermal treatments are easily applied to materials and the processing requirements are an oven, a pressure pot, and different solutions that are inexpensive.

Calcium (Ca) ions are crucial for cell-to-substrate interactions [12]. Moreover, our previous study demonstrated that the calcium chloride (CaCl_2_) hydrothermal treatment of a Ti implant impacted on epithelial sealing [13]. In addition, zinc (Zn) showed itself to be effective in promoting osteoblast proliferation [14], inhibiting bone resorption by decreasing osteoclastogenesis [15]. Moreover, Zn was found to be effective in enhancing laminin and collagen type IV, which is important for the assembly of the basement membrane [16]. The hydrothermal treatment of Ti with divalent strontium (Sr) can accelerate the process of osteogenesis [17]. Therefore, in the present study, we hypothesize that the hydrothermal treatment of Ti with Ca and Zn divalent cation solutions may promote the regeneration of epithelial tissue around the Ti implant. The aim of this study is to evaluate the efficacy of the hydrothermal treatment of Ti with the divalent cations of Ca, Zn, and Sr for epithelial tissue sealing and resistance to exogenous penetration.

## 2. Materials and Methods

### 2.1. Hydrothermal Treatment of Ti Plates and Implants

**Figure 1:**
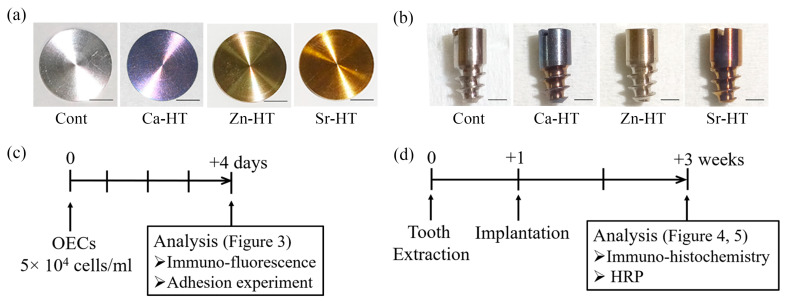
(**a**) Photographs of the control (Cont), calcium hydrothermally treated (Ca-HT), Zn-HT, and Sr-HT group plates (bar = 2 mm). (**b**) Photographs of the Cont and experimental groups’ Ti implants: Cont, Ca-HT, Zn-HT, and Sr-HT groups (bar = 2 mm). (**c**) Experimental protocol for the in vitro study. Rat oral epithelial cells (OECs) were analyzed for changes in the cell morphology 4 days after seeding on the samples. (**d**) Experimental protocol for the in vivo study. The implants were placed 1 week after tooth extraction. The structure of the peri-implant epithelial tissue around the implant was observed after 2 weeks of implantation.

Commercially pure titanium plates (15 mm diameter, 1 mm thickness, Japan Industrial Specification Class 1, H 4600, 99.9 mass %, Figure 1a) with a mirror-polished surface were used for the in vitro study. For the in vivo study, screw-shaped, pure titanium implants (2 mm diameter, 4.5 mm in length, 2.5 mm transmucosal, and 2 mm intrabony; Japan Industrial Specification Class 1, equivalent for ASTM grade I; Sky Blue, Fukuoka, Japan, Figure 1b) with a machined surface were utilized. Hydrothermal treatment, which requires an oven, a pressure pot (including a hydrothermal unit), and different solutions, was applied in the present study. The plates and screw-shape implants were washed in an ultrasonic bath with 100% acetone, distilled water, and 99.5% ethanol before hydrothermal treatment. There were four experimental groups: (1) the control (Cont) group of Ti samples without hydrothermal treatment; (2) the calcium hydrothermally treated (Ca-HT) group of Ti samples that were treated at 200 °C for 24 h with 15 mL of a 10 mmol/L aqueous solution of calcium chloride (CaCl_2_) in a hydrothermal unit (HU-50, SAN-AI Kagaku, Nagoya, Japan), as previously investigated [11,18,19]; (3) the Zn-HT group of Ti samples treated at 200 °C for 24 h with 15 mL of a 10 mmol/L aqueous solution of zinc chloride (ZnCl_2_) in a hydrothermal unit and; (4) the Sr-HT group of Ti samples treated at 200 °C for 24 h with 15 mL of a 10 mmol/L aqueous solution of strontium chloride (SrCl_2_) in a hydrothermal unit. To prevent surface contamination of the samples, all samples were washed with distilled water and stored under vacuum after treatment.

### 2.2. X-ray Photoelectron Spectroscopy Analysis of the Surface Characteristics

The surface of the experimental samples was analyzed by X-ray photoelectron spectroscopy (XPS) (K-alpha, ThermoFisher Scientific, East Grinstead, UK). All binding energies were referenced to the carbon 1s component at 285.0 eV. Overlapping peak images in the XPS spectra were separated by a computer-aided method.

### 2.3. Epithelial Cell Culture

An oral epithelial cell (OEC) culture was previously described [20]. Briefly, oral mucosa isolated from 4-day-old Wistar rats was incubated with dispase (1 × 10^3^ IU/mL) dissolved in Mg^2+^ and Ca^2+^-free phosphate-buffered saline (PBS) at 4 °C for 12 h. The oral epithelium was then isolated individually and dispersed by pipetting 10 times. The solution was filtered by a 40 µm cell strainer and directly seeded on the Ti plates. OECs were then cultured in defined keratinocyte serum free medium (DK-SFM; Gibco, Grand Island, NY, USA) and were grown in a humidified atmosphere of 5% CO_2_ at 37 °C. Immunofluorescence staining and adhesion of the OECs were analyzed 4 days after seeding (Figure 1c).

### 2.4. Immunofluorescence Staining for Adhesion Proteins

After 4 days of culture, OECs seeded on Ti plates were fixed with 4% paraformaldehyde for 10 min and pretreated with 0.5% Triton X-100 (Novocastra Laboratories, Newcastle-upon-Tyne, UK) for 3 min. The samples were then incubated with a polyclonal mouse anti-rat Ln-332 antibody (1:100 dilution, Santa Cruz Biotechnology, CA, USA) overnight at 4 °C. Samples were subsequently incubated with a fluorescein isothiocyanate (FITC)-labeled secondary antibody (1:100 dilution; Chemicon International, Billerica, MA, USA) at 37 °C for 2 h. Actin filaments were stained with tetramethylrhodamine isothiocyanate (TRITC)-conjugated phalloidin (1:100 dilution, Sigma-Aldrich, St. Louis, MO, USA) at 37 °C for 1 h. Imaging was performed using fluorescence microscopy (BZ-9000; Keyence, Osaka, Japan).

### 2.5. OEC Adhesion Evaluation

OEC adhesion assays were conducted as described in our previous report [18]. Briefly, non- or weakly attached OECs were removed by shaking (5 min × 3 at 75 rpm) using a rotary shaker (NX-20, Nissin, Tokyo, Japan) in DK-SFM medium. Adherent cells were measured as a percentage of the initial count. Attached OECs were counted by using a cell count kit (Cell Count Reagent SF, Nacalai Tesque, Kyoto, Japan). Absorbance at 450 nm was measured using a spectrophotometer (NJ-2300, Biotech, Tokyo, Japan) after the addition of a cell count reagent for 2 h at 37 °C.

### 2.6. Animals and Implantation

Male Wistar rats (5 weeks old, each weighing 120–130 g, n = 8 for each group) were used for all groups according to the animal care guidelines established by Kyushu University (A29-227-0). A required sample size of eight was determined by performing a power analysis based on our previous studies [21,22].

Our previous studies outline the implantation details using Wistar rats [23,24]. Briefly, under systemic and local anesthesia, the first molar of the right maxillary was extracted and, one week later, the experimental screw-shaped titanium implant was screwed into the extraction socket. After extraction and implantation, each rat was injected with buprenorphine (0.05 mg/kg) intramuscularly to alleviate suffering during surgery. The experimental rats were given powdered food and water and were maintained in a temperature-controlled room until euthanasia 2 weeks after implantation (Figure 1d).

### 2.7. Tissue Preparation and Immunohistochemistry

As previously described in our study [25], the rats were sacrificed and perfused with 4% paraformaldehyde fixative followed by an immersion in 10% ethylene diamine tetra-acetic acid (EDTA) at 4 °C for 3 days. The peri-implant tissue or periodontal oral mucosa was peeled from the right maxilla or implant and these isolated soft tissue samples were immersed in a 20% saccharose solution at 4 °C for 1 day followed by a 30% saccharose solution at 4 °C for 1 day. The samples were then inserted in O.C.T compound (Sakura Finetek, Tokyo, Japan) at 4 °C for 2 h, and then the samples were cut into 10-µm thick palate-buccal sections using a cryostat cooled to −20 °C. The avidin–biotin complex (ABC) procedure used for immunohistochemical staining was previously described [26]. The peroxidase transformed diaminobenzidine (DAB, ABC-DAB; Vector Laboratories, CA, USA) into a dark brown precipitant, revealing the localization of Ln-332. After lightly counterstaining with hematoxylin, the sections were photographed.

### 2.8. Horseradish Peroxidase (HRP) Topical Application

To assess the peri-implant epithelial sealing capacity, horseradish peroxidase (HRP) (50 mg/mL, type11, molecular weight approximately 40,000 Da; Sigma-Aldrich), a penetrative exogenous substance, was applied topically around the implant 2 weeks after implantation. The procedure of the HRP application was similar to that in our previous study [27]. Briefly, cotton balls were immersed in HRP dissolved in a buffered saline and the cotton was placed at the gingival margin around the experimental implant as gently as possible to prevent damage to the interface between the implant and gingiva. The HRP solution was continuously dripped onto the cotton balls every 10 min for 60 min.

### 2.9. Histochemistry of HRP

The rats were euthanized after 60 min and the samples were treated the same as the immunohistochemistry described in Section 2.7. Several 10-µm-thick sections were rinsed with 0.01 M PBS followed by incubation in a peroxidase staining DAB kit (Nacalai Tesque) for 30 min at room temperature. To evaluate the localization of HRP penetration, all sections were observed using light microscopy and the distance between the top of the peri-implant sulcular epithelium and the bottom of the staining was measured by linear measurements.

### 2.10. Statistical Analysis

Our experiment used 8 samples in each group, and an a priori Shapiro–Wilk test was performed to test for normality. One-way analysis of variance (ANOVA) with Scheffe’s post hoc was performed. Values of *p* < 0.05 were considered to be statistically significant. Data are indicated as the means ± standard deviation (SD). The statistical analysis (including 95% confidence intervals (95% CI)) was carried out with SPSS Statistics 19 software (IBM, Armonk, NY, USA).

## 3. Results

### 3.1. Surface Characteristics of the Specimens

**Figure 2:**
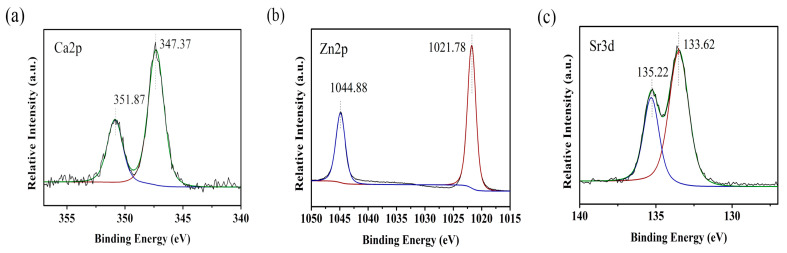
X-ray photoelectron spectroscopy (XPS) spectra of the Ca-HT, Zn-HT, and Sr-HT Ti plates. (**a**) Ca2p peaks were observed between 340 eV and 360 eV (Ca). (**b**) The Zn2p peaks were observed between 1015 eV and 1050 eV (Zn). (**c**) The Sr3d peaks were observed between 130 and 140 eV (Sr).

The Ca, Zn, and Sr spectra on the surface of the Ca-HT, Zn-HT, and Sr-HT Ti plates are shown in Figure 2, with clear Ca2p, and Zn2p, and Sr3d peaks observed in the three experimental groups.

### 3.2. Expression of Adhesion-Related Proteins in the Immunofluorescence Staining

**Figure 3:**
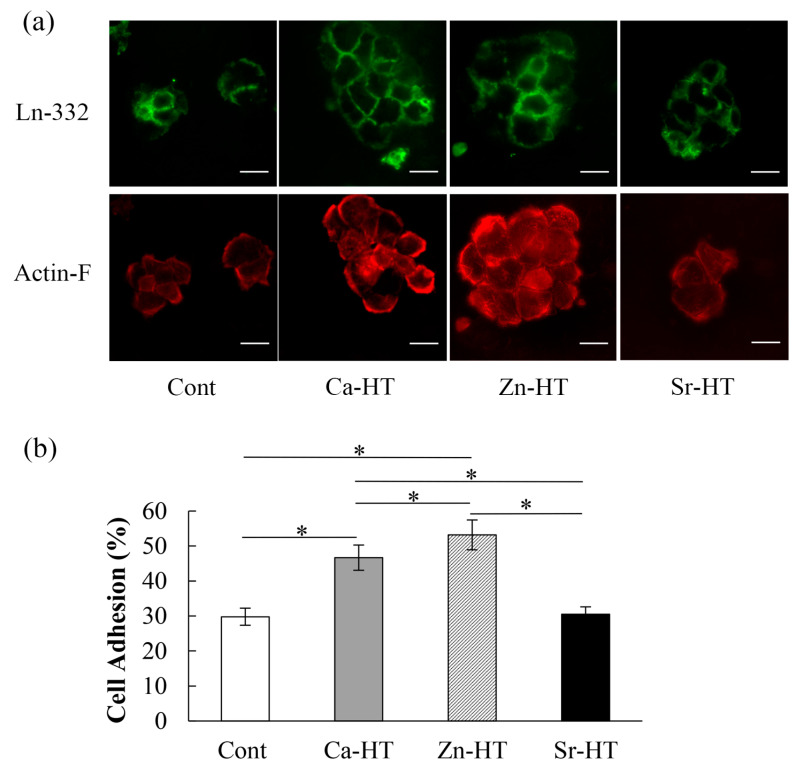
Adhesion of OECs to Cont, Ca-HT, Zn-HT, and Sr-HT Ti plates. (**a**) Localization of the adhesion-related proteins in OECs on the Cont, Ca-HT, Zn-HT, and Sr-HT Ti plates. Plates were incubated with mouse anti-rat laminin-332 (Ln-332) IgG followed by a fluorescein isothiocyanate-conjugated anti-mouse IgG secondary antibody (green). Actin filaments (Actin-F) were labeled with tetramethylrhodamine isothiocyanate-conjugated phalloidin (red) (bar = 40 μm). (**b**) The OEC adhesion ratio. Each bar represents the mean ± SD. One-way ANOVA with Scheffe’s post-hoc test; * *p* < 0.05 between the indicated groups.

The immunoreactive expression of Ln-332 in the Cont and experimental groups is shown in Figure 3a. The diffusely scattered signals of the Ln-332 immunoreactive area of the epithelial cytoplasm in the Zn-HT group were stronger than those in the other groups. The Ln-332 expression was nearly the same in the Ca-HT group compared with the Zn-HT group. Additionally, more actin filaments were observed in the OECs on Zn-HT than in the other groups. The actin filaments were weakly developed in the Cont and Sr-HT groups.

### 3.3. OEC Adhesion Assay

The OEC adhesion rates (%) of Cont, Ca-HT, Zn-HT, and Sr-HT groups were, respectively, 29.78 ± 2.43 (95% CI = 28.23–31.33), 46.66 ± 3.61 (95% CI = 44.04–48.27), 53.18 ± 4.27 (95% CI = 50.46–55.89), and 30.50 ± 2.10 (95% CI = 29.47–32.19). The Zn-HT and Ca-HT groups exhibited more adhered OECs than the Sr-HT and Cont groups that were statistically the same (Figure 3b).

### 3.4. Localization of Ln-332 in the Peri-Implant Epithelium (PIE)

**Figure 4:**
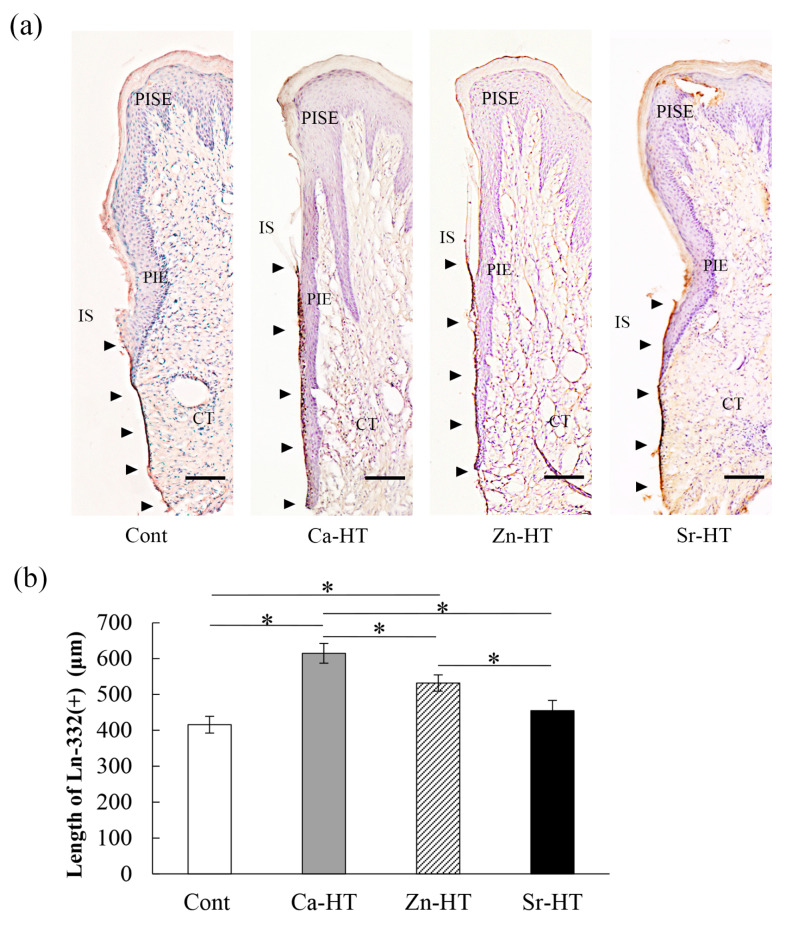
Immunohistochemical micrographs of Ln-332 localization in the peri-implant epithelium of the Cont, Ca-HT, Zn-HT, and Sr-HT groups after 2 weeks. (**a**) Arrowheads indicate the positive area of Ln-332 staining around the Cont, Ca-HT, Zn-HT, and Sr-HT implants (bar = 100 μm). IS, implant space; CT, connective tissue; PIE, peri-implant epithelium; PISE, peri-implant sulcular epithelium. (**b**) Length of the Ln-332 positive area on the samples. One-way ANOVA with Scheffe’s post hoc test; * *p* < 0.05 between the indicated groups.

The Ln-332 expression was observed as a band along the interface between the implant and PIE (Figure 4a). A previous study demonstrated that the interface between the dental implant and PIE can be divided into three portions: upper, middle, and lower [28]. The length of Ln-332 (μm) in Cont, Ca-HT, Zn-HT, and Sr-HT groups was, respectively, 415.46 ± 8.26 (95% CI = 395.92–435.00), 609.87 ± 10.87 (95% CI = 584.17–635.58), 563.07 ± 11.02 (95% CI = 537.01–589.15), and 454.69 ± 10.01 (95% CI = 430.84–478.54). In the Ca-HT and Zn-HT groups, Ln-positive staining was along the implant–PIE interface in the lower portion and even expanded into the middle portion. However, in the Sr-HT group, the length of Ln-positive staining was limited compared with the other two experimental groups. In addition, the length of the Ln-positive staining band in the Cont group was not obvious and was restricted to the lower area; this was significantly different in the Ca-HT and Zn-HT groups (Figure 4b).

### 3.5. Evaluation of HRP Penetration

**Figure 5:**
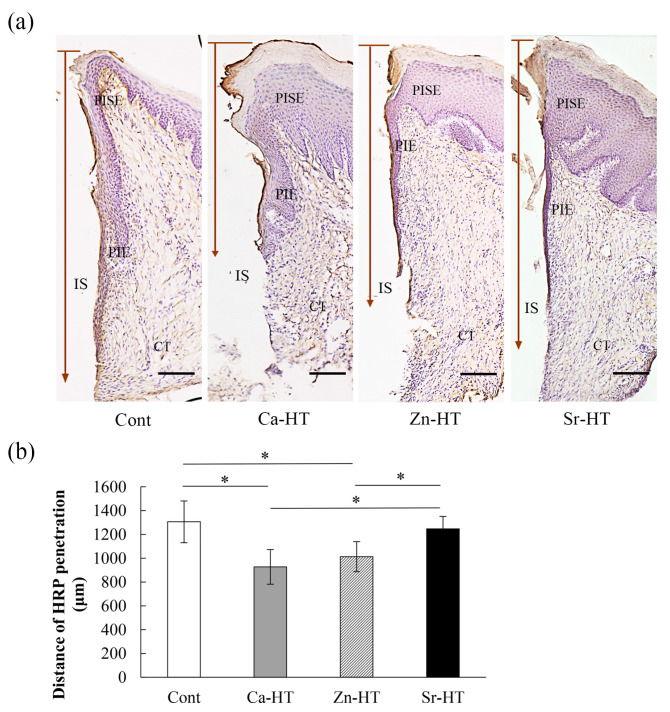
Horseradish Peroxidase (HRP) penetration of the peri-implant structure around Cont and the experimental implants 2 weeks after implantation. (**a**) Localization of HRP penetrating the peri-implant structure of the samples. The length of the arrow indicates the depth of the HRP penetration (bar = 150 μm). IS, implant space; CT, connective tissue; PIE, peri-implant epithelium; PISE, peri-implant sulcular epithelium. (**b**) The HRP penetration depth distance 2 weeks after implantation. One-way ANOVA with Scheffe’s post hoc test; * *p* < 0.05 between the indicated groups.

HRP was used for evaluating the sealing ability of the epithelial attachment around the Cont and experimental implant groups. The distance of HRP penetration (μm) in the Cont, Ca-HT, Zn-HT, and Sr-HT groups was, respectively, 1305.62 ± 61.95 (95% CI = 1159.13–1452.11), 927.38 ± 51.46 (95% CI = 805.70–1049.05), 1013.13 ± 44.66 (95% CI = 907.53–1118.72), and 1246.63 ± 36.85 (95% CI = 1162.50–1333.76). The distinct HRP reactions were limited to nearly the same area in the apical and middle portion of the epithelial attachment around the Ca-HT and Zn-HT group implants (Figure 5a). However, in the Cont group, the HRP reaction penetrated the epithelial layer and deeper into the connective tissue. Among the three experimental groups, the HRP reactions in the Sr-HT group invaded more deeply than in the other two experimental groups but had almost the same penetration compared to the Cont group (Figure 5b).

## 4. Discussion

In this study, hydrothermal treatments were performed to introduce Ca, Zn, or Sr on the surface of Ti. A previous study indicated that the hydrothermal treatment, regardless of the cation type, could enrich the surface with hydroxyl groups [18]. Another study demonstrated that the hydroxyl groups of the Ti surface could significantly enhance protein adsorption [29]. In addition, the adsorption of the adhesion protein can be promoted on a hydrophilic surface through the attachment of hydroxyl groups on the Ti surface [30,31]. Thus, we chose a hydrothermal treatment as an easy and inexpensive procedure to ameliorate epithelium adhesion to Ti implants. As previously demonstrated, a rougher surface has a negative effect on the adhesion of oral epithelial sealing [10]. However, with the hydrothermal treatment, we easily generated a divalent cation-rich outer surface with minimal surface topographical alterations [19]. This treatment may facilitate oral epithelial tissue cell adhesion to Ti implants.

The state of Ca, Zn, or Sr on the surface of Ti needs to be elucidated (i.e., whether they are ions, crystals, etc.). However, as our first in vitro experiment demonstrated (Figure 2), we detected Ca, Zn, and Sr peaks on the Ti surface. In our previous study, a calcium-related crystal structure was not observed using thin-film X-ray diffractometry [32]. Other studies have demonstrated that Zn- and Sr-HT of Ti implants also release ions [33,34]. Therefore, the state of the Ca, Zn, and Sr on the hydrothermally treated Ti implants could be a coupling scheme between Ti and these divalent cations, which may easily be released from the Ti surface. We then investigated the hydrothermal treatment of Ti with these aqueous divalent cations to facilitate PIE sealing around the implant.

Ln is a crucial protein known for its great wound healing ability in the formation of epithelium after implantation. Basal lamina attaches epithelial cells to the implant surface through HD. In addition, epithelial cells can produce Ln that enhances adhesion between epithelial cells and a substrate [35]. A previous study demonstrated that Ln can induce epithelial cell migration via integrins at the leading edge [36]. Additionally, Ln can also accelerate adhesion cells via integrin α6β4 of the regenerating oral mucosa [37]. Therefore, Ln localization can be an evaluation of early epithelial sealing and can be critical to promoting cell migration and healing of an epithelial wound [6].

Zn is one of the most prominent elements in wound healing modulation and is an important component in cell physiology [38]. Moreover, Zn^2+^ has the biggest impact of enhancing laminin binding, which is crucial to regulating specific cellular functions [16]. A previous study has shown that Madin-Darby Canine Kidney (MDCK) cells, an epithelial cell from canine kidneys, can immediately secrete extracellular matrix proteins, such as Ln-332, after seeding onto a substrate [39], and another study demonstrated that Zn can promote actin filament immigration in the MDCK cells, which accelerates the epithelial cells’ adhesion ability [40]. As a confirmation, in this study, the Zn-HT group indicated the strongest expression of Ln-332 and actin-F with immunofluorescence staining and the best adhesion of epithelial cells, even better than the Ca-HT group (Figure 3a,b). Moreover, the localization of the Ln-332 immunohistochemical staining area on Zn-HT was longer compared with the Cont and Sr-HT groups at the PIE–implant interface (Figure 4b). Thus, the hydrothermal treatment with Ca and Zn could provide better epithelial attachment to a Ti implant surface in PIE sealing.

Previous studies have shown that a binding molecule, heparan-sulfate proteoglycan (HSPG), may play a crucial role between the Ti implant and epithelium when Ti implants penetrate the oral mucosa and adsorb Ca ions on the Ti surface [18]. HSPG is also regarded as a binding mediator between adhesion proteins (such as Ln) on the epithelial cell membrane [41,42]. Our previous study also showed that positively charged Ca^2+^ may adsorb instantly onto the Ti surface via minus hydroxyl groups upon implantation [13]. Subsequently, HSPG might adsorb the Ca ions in the Ca-HT of Ti, which enhanced cell attachment on the Ti surface.

The Sr-HT group indicated limited Ln-332 localization and weak inhibition of HRP penetration compared with the other two experiment groups. Previous research reported that hydrothermal treatment with SrCl_2_ could promote osteogenesis [34]. However, Sr^2+^ has no significant effect on HSPG translocation [43], which indirectly means that Sr ions released from the Ti surface may not adsorb the HSPG more strongly or quickly than the Ca ion release. Thus, the hydrothermal treatment with Sr may not significantly accelerate epithelial sealing compared with the Ca-HT and Zn-HT groups.

In this study, we used the HRP penetration analysis to easily visualize the histology. The molecular size of HRP is approximately 40,000 Da, which is similar to that of lipopolysaccharides, and HRP has been diffusely applied as an alternative for endotoxins. The optical HRP penetration distance could be correlated to the strength of the epithelial sealing [27]. Indeed, the HRP penetration of the Zn-HT group was deeper than that of the Ca-HT group; however, the penetration was obviously shorter than the Cont and Sr-HT groups (Figure 5b). The Zn-HT and Ca-HT groups had strong epithelium adhesion and secure sealing with the Ti implant surface, which could protect against external stimuli encountered in clinical applications.

There are some limitations of this study. The first limitation is that we only applied one time period and temperature to the hydrothermal treatment processing. Our previous study demonstrated that temperatures less than 200 °C and processing times less than 24 h resulted in less bone tissue adhesion to Ti [19]. Thus, we may choose a higher temperature or longer processing time for future treatments. Another limitation is that we only used a 2-week in vivo experiment to observe the epithelial sealing. Longer implant durations should establish whether the hydrothermal treatment with these divalent cations of the Ti surface improve long-term function.

## 5. Conclusions

Hydrothermal treatment of Ti with Zn and Ca ion aqueous solutions showed early peri-implant epithelial sealing after implantation. The epithelial sealing was not promoted by hydrothermal treatment of Ti with a Sr ion aqueous solution. This study indicates that the Zn and Ca ion hydrothermal treatments can provide Ti implants with protection against external oral stimuli.
